# Eleventh Annual Meeting of the Michigan Dental Association

**Published:** 1866-03

**Authors:** G. W. Stone


### Proceedings of Societies.

The eleventh annual meeting of the Michigan Dental Association was held Jan. 30th, 1866, at the Biddle House, in the city of Detroit, and was called to order by the President, Dr. Watling. On motion, adjourned until two o'clock.

Afternoon session—President in the chair—Constitution read by the Secretary.

Drs. Douglas, Benedict and Cahoon were appointed a committee on the order of discussions, who reported the following:

1st. Reading of Essays.

2d. The best means of improving the practice and elevating the profession of Dentistry.

3d. Anæsthetics—Their proper use and relative value.

4th. Extracting teeth—When it should be done and when not; the best instruments for the purpose, and the subsequent treatment when any is required.

5th. Absorption of Alveolar Process — Causes and Treatment.

6th. Filling teeth: The relative value of different materials and the mode of operating in different cases.

7th. The best method of obtaining accurate impressions and models of the mouth.

8th. The relative value of different materials as a base for artificial teeth.

9th. Neuralgia.

10th. Miscellaneous.

The following named gentlemen were ballotted for and elected members of this association, viz: Drs. W. H. Jackson, Ann Arbor; E. S. Holmes, Grand Rapids; Geo. Leary, Adrian; P. R. Hovey, Fentonville; J. C. J. Crooks, Birmingham: C. B. Stodard, Ann Arbor; J. C. Parker, Grand Rapids; Joseph Lathrop, Detroit.

Minutes of last regular meeting read and approved. Dr. Benedict from the committee to prepare a memorial to the regents of the university asking for the establishment of a dental chair in the university, reported that such a memorial had been prepared and presented to the regents. The committee procured the services of Dr. Taft, of Cincinnati, to present the subject to the regents. The latter referred the matter to the medical faculty who reported against the establishment of such a department at present, and the report was adopted by the regents. The report of the committee was adopted and the committee discharged. The association then proceeded to the election of officers for the ensuing year, with the following results:

President, Dr. G. L. Field, Detroit; Vice-President, J. A. Harris, Pontiac; Secretary, G. W. Stone, Albion; Treasurer, H. Benedict, Detroit. Drs. Harris, Cahoon and Bancroft Executive, Examining, and Publishing Committee.

The President elect on being conducted to the chair by Drs. Cahoon and Douglas, made a few appropriate remarks. The retiring President, Dr. Watling, delivered an interesting address, which was referred to the publishing committee.

On motion of Dr. Watling, a committee was appointed to arrange a regular order of business for the association. Drs. Watling and Stone were appointed such committee. The Treasurer submitted his annual report, showing a balance on hand of $48.46, and on motion, the report was adopted. The Secretary presented a bill of expenditures for the year of $8.66, which was ordered paid. On motion of Dr. Benedict, the Secretary was directed to draw an order on the Treasurer of $25 in favor of Dr. Taft for traveling expenses.

Dr. Harris exhibited an automatic mallet, in reference to an offer of a premium for the best mallet, and it was referred to a committee, consisting of Dr. Benedict, Cahoon and Stone. Adjourned to meet to-morrow morning at 9 o'clock.

MORNING SESSION, JAN. 31.

President in the chair — Minutes of last meeting read and approved. On motion of Dr. Benedict, Dr. Taft, of Cincinnati, and Drs. Chesebrough and Harroun, of Toledo, were elected honorary members of this association. A communication was received from Dr. C. B. Porter, of Ann Arbor, saying that he had retired from the practice of dentistry, and wished to resign his position as member of the association. On motion, his resignation was accepted, and his name placed on the list of honorary members. Dr. Watling offered the following resolution:

*Resolved*, that it be deemed derogatory to the best interest of the profession for any of its members to take students for a less term than three years, and under no circumstances, unless he pledge himself to attend two terms in some regular dental college.

After remarks from Dr. Bancroft, Taft, Douglas, Watling and Chesebrough, it was unanimously adopted.

Also the following was offered by Dr. Watling, which was unanimously adopted:

*Resolved*, that any person entering the profession after this date, shall not be eligible to membership in this association until he has graduated from some regular dental college. On motion of Dr. Warner, both of the above resolutions were incorporated in the by-laws of the association.

Dr. Harris presented a little girl with a supernumery tooth, and asked the opinion of the association in regard to extracting.

On motion of Dr. Warner, a committee consisting of Drs. Benedict and White was appointed to arrange for clinical operations.

Dr. Harris offered the following resolution, and on motion it was adopted:

*Resolved*, that the officers of this association and as many members thereof as may be necessary, be a committee to obtain a charter for and incorporate this association.

The compliments of Mr. Fred. Stearns were received by the association inviting the members to his house this (Wednesday) evening, and, on motion, the invitation was accepted.

Dr. Harroun, of Toledo, said he was commissioned, by the dental association of Northern Ohio, to send greeting to this association. The President responded in fitting terms. On motion of Dr. Knowlton, of Detroit, the Northern Ohio Dental Association, and all other similar bodies in Ohio, were invited to meet with this association at its next annual session, either by delegation or otherwise.

Dr. Holmes, of Grand Rapids, presented the following, and on motion it was adopted:

*Resolved*, that a committee of three be appointed to take into consideration the propriety of adopting a standard of elegibility to membership in this association, and to report the result of their deliberations at this meeting. The chair appointed Drs. Holmes, Benedict and Watling such committee. Dr. Chesebrough read a paper on the "Duty of the Dental Profession," showing clearly the great responsibility of the dentist to his patrons.

Adjourned until two o'clock.

### AFTERNOON SESSION.

Dr. G. B. Snow, of Buffalo, was elected an honorary member of this association.

On motion, the paper read by Dr. Chesebrough was ordered to be printed.

The committee appointed to arrange an order of business for this association reported the following:

1. Reading the Constitution.
2. Admission of new Members.
3. Reading the minutes of previous meetings.
4. Report of Officers and Standing Committees.
5. Election of Officers.
6. Installation of Officers.
7. Retiring President's Address.
8. Miscellaneous Business.

On motion, the report was adopted and ordered incorporated in the by-laws of the association.

Dr. Chesebrough exhibited some fine specimens of fœtal development. Dr. Robinson, of Jackson, read a paper entitled "Little Things," which was received with much favor. Also one by Dr. Douglass, both of which were referred to the publishing committee.

Dr. Benedict read a paper containing a summary of his work for eighteen months, and gave some incidents of preserving teeth after they were badly decayed and nerve dead; the paper was referred to the publishing committee.

Dr. Field read a paper treating of "Dental Societies and their influence," which was received with much applause and referred to the publishing committee.

Dr. Taft being obliged to leave soon, Dr. Robinson requested him to give his treatment for the cure of alveolar abscess where it discharged down the fang of the tooth. Dr. Taft said that the treatment would be modified to a very great extent, by the condition of the health of the patient. The first thing to be done is to remove the cause of irritation; and then reach the point of disease through the most feasible channel. Open up through the fang of the tooth; clear out the canal; see that no foreign substance is lodged there, in other cases trepanning or cutting through the process to the apex, removing all the dead bone and treating the system with alteratives and tonics. Several other members spoke upon the subject, refering to patients under their charge, whom they treated in a manner similar to that recommended by Dr. Taft.

Adjourned until to morrow morning at 9 o'clock.

MORNING SESSION, FEB. 1ST.

Minutes read and approved. Committee on qualifications for membership submitted the following qualifications necessary to become a member of the association:

1. Good moral character—evidence to be presented by two reputable persons.

2. Honorable, manly, and friendly feelings and conduct towards the members of the profession.

3. A determination to practice dentistry as an art founded upon science, or as a liberal profession.

4. Must be considered a reputable practitioner of dental surgery by the members of the profession who are acquainted with him.

5. Must be a graduate of a dental college or a graduate of a medical college, and, for at least one year, a regular practitioner of dentistry; or if not a graduate, must have been a respectable practitioner of dentistry for at least six years, and if the candidate commence practice after this date, he must invariably be a graduate of a dental college.

On motion, the report was adopted.

The subject of anæsthetics was then taken up, and discussed in regard to their proper use and relative value.

Dr. Lathrop spoke in favor of nitrous oxide; would not give it when the heart is diseased; had used it over two years, during which time had administered it to from 6 to 8 hundred patients; never saw any injurious effects from the use of it; gave 36 gallons at one time to a lady without any bad symptoms; produces perfect anæsthesia.

Dr. Field—Prefers chloroform to any other agent; had tried nitrous oxide and found no particular objection to it; thinks the bad effect would be *felt* immediately, if ever; thinks the bad effect of chloroform is from the impurity of the article used; is now using a pure article prepared by Dr. Duffield, of Detroit, without producing nausea or debility; gives it on an empty stomach; enforces on the mind of his patients the necessity of obeying him in every particular during the operation.

Dr. Cahoon—Likes the use of nitrous oxide on account of its safety, but does not use it as it is too troublesome and expensive.

Dr. Knowlton—Likes nitrous oxide better than any other agent; thinks it is perfectly safe.

Dr. Farmer—Thinks nitrous oxide no safer than chloroform; prefers the latter.

The subject of extracting teeth was next taken up.

The Secretary believed the tendency among the dental profession was to save too many teeth that should, in view of the health of the patient, be removed. He would guard against riding a hobby, either on one side or another, and said to allow certain teeth to remain was detrimental to the health of the patient.

Dr. Cahoon—Would save as many natural teeth as possible, and would not extract any if the patient would submit to have them filled.

Dr. Holmes coincided with Dr. Cahoon. Many members participated in the discussion, arguing generally in favor of saving all the natural teeth.

Adjourned until two o'clock.

### AFTERNOON SESSION.

A great portion of the afternoon was occupied in clinical operations, after which the association discussed the question of filling teeth, and the relative value of the various materials used. On motion, it was resolved to hold the next annual meeting of the association at Adrian. On motion of Dr. Watling, the executive committe was instructed to arrange an order for discussion for the next annual meeting, and send it to the Secretary, who is instructed to have it printed on the circulars of invitation.

Adjourned to meet again at 7 o'clock in the evening.

### EVENING SESSION.

Committee on automatic mallet excused from reporting till the next meeting. Drs. Ban-croft, Holmes, White, Field, Warner, W. H. Jackson and Farmer were elected delegates to the American Dental Association, to be held at Boston in July next.

Dr. Stodard, of Ann Arbor, being elected in violation of constitution, a motion was made to return him the fee, and inform him of the illegality of the proceedings, which motion was laid on the table until the next annual meeting of the association.

Dr. Farmer asked for some views upon the use of chromic acid, upon which Dr. Field stated that he used it with great success. He had a patient who could not bear the least touch of the instrument upon a tooth, and he procured some chromic acid and used it, by way of experiment, after which he excavated freely without producing the least pain to the patient; said he would not do without it.

Dr. Snow had used it several times, but was not satisfied with the result; thought it produced as much pain as chloride of zinc. Dr. Holmes had used chromic acid a few times; was pleased with the result, although it produced severe pain for a few moments, afterwards the tooth could be excavated without hurting the patient; is afraid that it injures the tooth, but thus far has seen no bad effect.

On motion of Dr. Benedict, a committee of three was appointed to memorialize the regents of the university for the establishment of a dental chair in the university. The association appointed Drs. Benedict, Stone and Field as such committee.

Dr. Holmes addressed the association on the subject of dental literature, and urged the neceessity of subscribing for and reading as many dental periodicals as possible. On motion, Mr. Fred. Stearns was elected an honorary member of the association. A vote of thanks was tendered to Mr. A. F. Tablor for the gratuitous use of a room in the Biddle House, in which the sessions of the association were held. Also to the press for the publication of the proceedings of the association. The remainder of the session was spent in a jovial, friendly discussion of various subjects connected with the profession. On motion, adjourned to meet at Adrian on the 4th Tuesday in January, 1867.

G. W. STONE, Cor. and Rec. Sec'y.

